# Young adult non-smokers’ exposure to real-world tobacco marketing: results of an ecological momentary assessment pilot study

**DOI:** 10.1186/s13104-017-2758-7

**Published:** 2017-08-31

**Authors:** Shyanika W. Rose, Andrew Anesetti-Rothermel, Hoda Elmasry, Ray Niaura

**Affiliations:** 1 Schroeder Institute at Truth Initiative, Washington, DC USA; 2 College of Global Public Health, New York University, New York, NY USA

**Keywords:** District of Columbia, Marketing, Pilot projects, Tobacco, Young adult, Health equity

## Abstract

**Background:**

The aims of this pilot study were to assess and characterize non-current smoking young adults’ exposure to tobacco marketing through an ecological momentary assessment protocol.

**Methods:**

Ecological momentary assessment (EMA) consists of repeated measurement of momentary phenomena and is well-suited to capture sporadic experiences in the real-world, such as exposure to tobacco marketing. EMA has the potential to capture detailed information about real-world marketing exposures in ways that reduce recall bias and increase ecological validity. In this study, young adults (n = 31; ages 18–25) responded to random prompts regarding their momentary exposure to tobacco marketing via text messages on their smartphones for 14 days (n = 1798 observations). Unadjusted and adjusted analyses were conducted using multilevel logistic regression to assess the odds of exposure accounting for correlation of multiple repeated measures within individuals while controlling for variability between individuals.

**Results:**

Respondents reported, on average, two momentary exposures to tobacco advertising in the 14-day study period. In adjusted analyses, African–American (aOR 3.36; 95% CI 1.07, 10.54) and Hispanic respondents (aOR 5.08; 95% CI 1.28, 20.13) were more likely to report exposure to tobacco advertising. Respondents were also more likely to report exposure when also exposed to others using tobacco products and when they were at stores compared with at home (aOR 14.82; 95% CI 3.61, 60.88).

**Conclusion:**

Non-smoking young adults report exposure to tobacco marketing particularly at the point-of-sale, with the highest likelihood of exposure among African-American and Hispanic young people. EMA protocols can be effective in assessing the potential impact of point-of-sale tobacco marketing on young adults.

**Electronic supplementary material:**

The online version of this article (doi:10.1186/s13104-017-2758-7) contains supplementary material, which is available to authorized users.

## Background

Exposure to tobacco marketing has been associated with increased risk of smoking in youth [1, 2] and young adults [3, 4]. Individual exposures throughout time and places are cumulative and can change positive expectancies and normative beliefs about tobacco products [5]. Exposure to tobacco marketing in real world settings affects many facets of tobacco use, but its role in tobacco use initiation among non-users is not well understood [6, 7]. Exposure to tobacco marketing may also be more prevalent among minority populations. For instance, there are significantly more menthol advertisements in areas with higher proportions of African–American residents [8, 9] and near schools with more African American students [10]. Exposure to tobacco marketing and brand recognition is higher among non-smoking African–American [11, 12] and Hispanic [12] youth and young adults and tends to be associated with greater risk of smoking initiation. Identifying how young adult non-smokers are exposed to tobacco marketing can help to understand who is at risk of exposure and potentially future tobacco use.

This study utilized a real-time and ecologically valid measure of exposure to tobacco advertising through ecological momentary assessment (EMA) [13, 14]. EMA can provide real-time tracking of exposure to and attitudes towards tobacco advertising closer to the moment of exposure, rather than through retrospective recall [1, 15, 16]. Prior EMA studies have examined self-reported overall tobacco advertising exposure [3, 17] or exposure to POS tobacco outlets [18] However, few studies have focused on exposure of non-current smokers to tobacco marketing. One study of college students found that almost 70% of self-reported exposure to tobacco marketing measured through an EMA protocol was at point of sale [19], and that exposure was related to future risk of smoking among both smokers and non-smokers [3]. The current study tested a protocol to assess real time exposure to tobacco advertising through EMA and identified correlates of exposure.

## Methods

### Ethical considerations

The study was approved by the Chesapeake institutional review board (Study Number #Pro00012367). Respondents were provided with informed consent to participate and identifiable information was kept separate from respondent survey and EMA text message responses. The EMA data was transmitted via standard text messages (SMS) via a respondents’ smartphone to a secure database. In the EMA, respondents were simply asked to provide numeric responses to questions without providing other identifiable information. In transit, SMS messages are encrypted. Once received, the data were stored in a secure firewall-protected and password-protected database via an encrypted representational state transfer (REST) application programing interface with access only to authorized users.

### Recruitment

As part of an EMA pilot study for young adult non-smokers that also examined store visiting behavior (not reported in this study), participants were recruited in summer 2015 via online and print advertisements (e.g., Craigslist, local newspaper, and flyers). In total, 258 individuals responded to the screener within a 1-month period. Eligible respondents were (1) between the ages of 18–25, (2) did not smoke cigarettes in the past 30 days, (3) lived and worked/attended school in Washington, DC, (4) owned their own android or iPhone smartphone with an unlimited text message plan, and (5) agreed to participate in the study (n = 31). This pilot protocol was developed to determine the feasibility of engaging young adult non-smokers in reporting on their exposure to tobacco marketing and store visiting building, in part, on an approach used in a prior study of smoker’s relapse and store visiting used by members of this study team [18].

### Data collection

All 31 participants completed a baseline survey of participant demographics, tobacco use history, and susceptibility to future cigarette use (see Additional file 1). Respondents downloaded the study application to their phone which enrolled them in a text message protocol. Over the next 14 days they were randomly prompted 6 times a day about their exposure to tobacco advertising. As a result, each respondent was assessed over 80 times. They had 1 h to respond to each prompt via text message before it expired. All 31 respondents completed the EMA period.

### Measures

Participant baseline measures included gender, race/ethnicity (White, African–American, Hispanic, other), past 30-day cigarette smoking status (y/n), age, self-reported personal financial status (live comfortably, meet needs with a little left, just meet basic needs, don’t meet basic needs), and smoking status as ever (even one puff) or never. They were also asked about their susceptibility to future cigarette use using the validated three-item susceptibility to smoke index measured on a four point scale of definitely yes, probably yes, probably no, and definitely no (“Do you think in the future you might experiment with cigarettes?,” “At any time in the next year do you think you will smoke a cigarette,” and “If one of your best friends were to offer you a cigarette, would you smoke it?”) [20]. Respondents who answered definitely no to all three questions were deemed non-susceptible, all others were deemed susceptible. EMA prompts measured tobacco marketing exposure as the outcome [“In your current location, do you see any advertisements or signs promoting tobacco?” (y/n)], current location (home, work, other’s home, bar/restaurant, vehicle/in transit, outside, store, other), and exposure to tobacco use (“Is anyone using tobacco products in your current location?” Yes, in view, yes, in my group, no) adapted from a prior study [17]. Participants self-defined what constituted tobacco marketing.

### Analysis

Descriptive statistics were used to characterize participants in terms of sociodemographic factors, smoking status and EMA responses. The analysis utilized multilevel logistic regression modeling using Stata version 13 in a two-level model, to account for clustering of multiple EMA responses within individual respondents. We anticipate that responses by the same individual are correlated and thus a multilevel modelling approach properly adjusts standard errors and associated confidence intervals. Additionally, multilevel models using repeated measure data allows for including all subjects’ observations despite the potential for missing data points for incomplete response to the EMA by participants. We modeled the likelihood of respondent momentary exposure to tobacco advertising when responding to the random prompts. Multilevel modeling extends to binary outcomes by using a logit link function, in which the log odds of momentary exposure to tobacco advertising was modeled as a linear combination of correlates. We first estimated the null model (a model with no predictors) finding that the logistic multilevel model was preferable to an ordinary logistic regression model based on a likelihood ratio test provided by STATA showing a highly significant result [χ^2^ (1) 28.99 p = 0.00]. We report 95% confidence intervals with significance levels set at the p < 0.05 level for all statistical analyses. Correlates of exposure included gender, race/ethnicity, financial situation, baseline cigarette susceptibility, location when responding to EMA prompts, and momentary exposure to smoking. We report both bivariate crude odds ratios and adjusted odds ratios in the multilevel logistic regression models along with 95% confidence intervals reporting significance at the p < 0.05 level. We retained all correlates in the adjusted model regardless of significance in the bivariate analyses to examine the potential joint effects of these variables on advertising exposure.

## Results

The respondents (Table 1) were predominantly female (74%) and African–American (58%). Respondents were highly educated with 61% having a Bachelor’s degree or higher and had a comfortable personal financial situation with 65% meeting needs with a little left or living comfortably. The majority (66%) had ever had even a puff of a cigarette but only one respondent had smoked 100 lifetime cigarettes. The remaining participants were never cigarette smokers. At baseline, 48% were susceptible to future cigarette use.

**Table 1:** Descriptive statistics of participant characteristics and ecological momentary assessments

	Mean/N	SD/%
Sociodemographics		
Mean age	22.5	1.9
Female	23	74.0
Race/ethnicity		
Non-Hispanic African American	18	58.1
Non-Hispanic White	9	29.0
Non-Hispanic Asian	2	6.5
Hispanic	2	6.5
Education		
Some college, no degree	11	35.5
Associate’s degree	1	3.2
Bachelor’s degree	18	58.1
Master’s degree	1	3.2
Financial situation		
Live comfortably	5	16.1
Meet needs with a little left	15	48.4
Just meet basic expenses	10	32.3
Don’t meet basic expenses	1	3.2
Smoking status^a^		
Never	11	35.5
Ever	20	65.5
EMA responses		
Completed	1798	70.2
Abandoned	83	3.2
Expired	674	26.3

^a^Smoking status defined as having ever tried at least one puff of a cigarette

During the EMA, the respondents were asked a total of 2560 random prompts, responding to 73% and completing 70%. The average number of complete random prompts was 58 per subject (median 59, range 25–75). On average, over the 14 day EMA period, respondents reported 1.9 exposures to tobacco advertising (range 0–10).

For the null multilevel model of exposure to tobacco advertising, the intraclass correlation was 0.24 indicating that 24% of the variance in tobacco advertising exposure was accounted for by clustering of observations within participants. In unadjusted analyses (Table 2), Hispanic respondents, participants not meeting basic financial needs, those exposed to tobacco use in their group or in their view, and respondents who were outside, at work or in a store had higher odds of being exposed to tobacco advertising.

**Table 2:** Unadjusted and adjusted multilevel logistic regression models of reported momentary exposure to tobacco advertising (N = 1794^a^)

Variable	%^b^	Unadjusted model			Adjusted model		
		OR	95% CI		aOR	95% CI	
Female	72.58	1.67	0.51	5.42	*3.92*	*1.10*	*13.96*
Race/ethnicity							
Hispanic	6.79	*8.01*	*1.45*	*44.21*	*5.08*	*1.28*	*20.13*
NH AA	58.51	2.94	0.94	9.17	*3.36*	*1.07*	*10.54*
NH Asian	6.90	0.63	0.05	8.13	1.60	0.14	18.29
White (ref)	27.81	–	–	–	–	–	–
Personal financial situation							
Live comfortably (ref)	16.52	–	–	–	–	–	–
Meet needs with a little left	46.55	0.95	0.27	3.35	0.47	0.15	1.46
Just meet basic needs	33.65	1.43	0.39	5.23	1.06	0.34	3.35
Don’t meet basic needs	3.28	*10.41*	*1.31*	*82.63*	*6.12*	*1.16*	*32.37*
Baseline susceptibility to future tobacco use							
Yes	47.89	1.32	0.51	3.45	1.73	0.74	4.06
No (ref)	52.11	–	–	–	–	–	–
Using tobacco							
Yes, in my group	2.89	*17.05*	*7.60*	*38.24*	*17.04*	*7.47*	*38.87*
Yes, in my view	4.62	*13.42*	*6.56*	*27.45*	*14.43*	*6.38*	*32.65*
No (ref)	92.27	–	–	–	–	–	–
Location							
Bar/restaurant	4.89	1.29	0.29	5.88	0.99	0.20	4.95
Other	4.45	1.22	0.27	5.56	0.80	0.16	4.01
Others home	7.17	1.58	0.41	6.07	0.95	0.23	4.01
Outside	4.73	*4.14*	*1.50*	*11.41*	1.46	0.45	4.77
Store	1.00	*10.66*	*2.50*	*45.53*	*14.82*	*3.61*	*60.88*
Vehicle/transit	7.17	2.18	0.88	5.42	1.17	0.42	3.21
Work	14.52	*2.46*	*1.06*	*5.67*	2.39	0.96	5.94
Home (ref)	56.06	–	–	–	–	–	–

Italic responses have 95% confidence intervals that do not include 1 and are significant at the p < 0.05 level of significance

^a^Number of observations in final fully adjusted model

^b^Of complete responses

Though based on small sample sizes, when adding covariates to the model (Table 2), African–Americans (aOR 3.36, 95% CI 1.07, 10.54) and Hispanics (aOR 5.08, 95% CI 1.28, 20.13) had higher odds of reporting momentary exposure to tobacco advertising than Whites. Asians were no more likely to report exposure than Whites. Respondents who did not meet financial needs compared with those who lived comfortably were more likely to report exposure to advertising (aOR 6.12, 95% CI 1.16, 32.37). Females also reported greater odds of exposure in adjusted analyses (aOR 3.92, 95% CI 1.10, 13.96). Respondents were more likely to report exposure to tobacco advertising if someone in their group was using tobacco (aOR 17.04, 95% CI 7.47, 38.87) or someone in their view was using tobacco (aOR 14.43, 95% CI 6.38, 32.65). There were no significant differences in odds of reporting exposure based on baseline susceptibility to future cigarette smoking. Finally, respondents had 15 times the odds of reporting exposure to tobacco advertising if they were at a store (aOR 14.82, 95% CI 3.61, 60.88) compared to when they were at home. In adjusted analyses, no other locations were significantly different than home.

## Discussion

When controlling for other factors, African American and Hispanic participants are more likely to report exposure to tobacco advertising. Respondents were also significantly more likely to report exposure to advertisements in stores. A large body of research has found increased tobacco advertising in stores in minority neighborhoods [8–10, 21, 22] suggesting that greater exposure among minority respondents may be due to more advertising in the environment. Point-of-sale tobacco marketing exposure may be particularly problematic, as exposure in stores, but not through other media, has been associated with increased future smoking risk [23]. Additionally, store type where respondents shop may play a role in these results, with convenience stores compared with other store types like grocery stores, more likely to have tobacco marketing [24]. We did not ask respondents who were at stores at EMA response to report the type of store visited, though this or automated geolocation monitoring through the smartphone could be added to future studies. However, though significant, these results should be interpreted cautiously and replicated in larger studies due to low sample sizes and wide confidence intervals.

Respondents were also more likely to report advertising exposure when in a group or in view of others using tobacco products. This finding is concerning as a prior EMA study of young adults found that being with a friend when exposed to pro-tobacco marketing was associated with greater intention to smoke in the future compared with exposure when alone, which was unrelated to intention [25]. Additionally, experiencing tobacco advertising in places where tobacco use is present may both serve as cues for use [26, 27]. Multiple consistent ‘social exposures’ (such as smoking and tobacco marketing) may interact to make tobacco use more normative which can in turn influence smoking risk [28].

### Strengths and limitations

This study has several limitations. The small sample size for the pilot study precludes broad generalizations and the study sample was predominately female; however, it did include a relatively diverse sample and is in line with the sample size in other pilot EMA studies of tobacco-related behaviors [25, 29, 30]. This study also focused on responses to random prompts, rather than also incorporating user-initiated responses as in other studies of marketing exposure [19, 25]. This approach may, therefore, have missed some advertising exposures, however, it reduces potential bias introduced by study-induced heightened participant awareness of tobacco marketing, which has been associated with increased susceptibility [31] and future use [16].

Strengths of the study are the wide applicability of the text messaging approach due to 85% smartphone ownership among young adults and 100% of text messaging use among smartphone owners in that age group [32]. Additionally, the response to the random prompts was relatively high, comparable with other studies of youth or young adults and generated nearly 60 observations on average per respondent [33, 34]. Momentary reporting of tobacco marketing exposure reduces recall bias and was more strongly correlated with future susceptibility than recall measures [19]. Additionally, the self-reported measure was broad-based in assessing marketing exposure through multiple channels and not just at the point-of-sale.

This pilot study finds that this EMA approach is feasible to engage young adult non-smokers in reporting on their momentary exposure to tobacco marketing. We intend to develop a larger study which examines differences in exposure between vulnerable population young adults and non-vulnerable young adults. This future study will assess whether differential neighborhood and individual exposure to tobacco marketing is associated with greater susceptibility to future use of tobacco, tobacco use disparities in vulnerable groups, and greater likelihood of tobacco use initiation over time.

## Conclusions

This pilot study shows that both susceptible and non-susceptible young adult nonsmokers are routinely exposed to tobacco marketing in their daily life, particularly at the point-of-sale. EMA protocols can be effective in assessing the potential impact of point-of-sale tobacco marketing on young adults. Regulations on point-of-sale marketing [35] may reduce exposure for young adults.

## Additional file

**Additional file 1: Appendix A.**Baseline study instrument.

## Abbreviations

aOR: adjusted odds ratio
CI: confidence interval
EMA: ecological momentary assessment
