# Supplementary material for: Young adult non-smokers’ exposure to real-world tobacco marketing: results of an ecological momentary assessment pilot study
Source: BMC Res Notes. 2017 Aug 31;10:435. doi: 10.1186/s13104-017-2758-7 (PMC5580291; doi:10.1186/s13104-017-2758-7)
Supplement: Supplementary file 1 — Additional file 1: Appendix A.Baseline study instrument. [file 13104_2017_2758_MOESM1_ESM.pdf]

TRAX (Tobacco Real-world Advertising eXposure) Study

Which of the following statements best describes you?

TRAX Study Baseline Survey

Thank you for your participation in the TRAX study. Please start the baseline survey now. All information will be kept confidential and will not be shared without your permission except as required by law. You may stop this survey at any time.

A. Demographics

Ethnicity

1. Male
2. Female

Ethnicity

1. Hispanic or Latino
2. Not Hispanic or Latino

Race:

1. White
2. Black or African American
3. Asian
4. Native Hawaiian or Other Pacific Islander
5. American Indian or Alaska Native

A1. What is the highest level of school you have completed?

1. No formal education
2. 1st, 2nd, 3rd, or 4th grade
3. 5th or 6th grade
4. 7th or 8th grade
5. 9th grade
6. 10th grade
7. 11th grade
8. 12th grade NO DIPLOMA
9. High school graduate – high school DIPLOMA or the equivalent (GED)
10. Some college, no degree
11. Associate’s degree
12. Bachelor’s degree
13. Master’s degree
14. Professional or Doctorate degree

A2. Are you currently enrolled in a degree program?

1. No
2. Yes, high school
3. Yes, vocational/technical school
4. Yes, community college or junior college
5. Yes, 4-year college or university
6. Yes, graduate or professional school
7. Yes, other

A4. We would like to get a better estimate of your total HOUSEHOLD income in the past 12 months before taxes. Was it…

1. Less than $5,000
2. 5,000 to 7,499
3. 7,500 to 9,999
4. 10,000 to 12,499
5. 12,500 to 14,999
6. 15,000 to 19,999
7. 20,000 to 24,999
8. 25,000 to 29,999
9. 30,000 to 34,999
10. 35,000 to 39,999
11. 40,000 to 49,999
12. 50,000 to 59,999
13. 60,000 to 74,999
14. 75,000 to 99,999
15. 100,000 to 149,999
16. 150,000 or more

A5. Considering your own income and the income from any other people who help you, how would you describe your overall personal financial situation? Would you say you:

1. Live comfortably
2. Meet needs with a little left
3. Just meet basic expenses
4. Don’t meet basic expenses

A6. Do you consider yourself to be…?

1. Heterosexual, or straight
2. Gay or lesbian
3. Bisexual
4. Transgender
5. Other (Please specify)_____________________

A6. What area of DC do you live in?

1. Northwest
2. Northeast
3. Southwest
4. Southeast

A7. What area of DC do you work or go to school in?

1. Northwest
2. Northeast
3. Southwest

4. Southeast

B. Tobacco Use

LYAC-R B1. Have you ever used or tried…? (For cigarettes, cigars, cigarillos, and electronic cigarettes, this includes "even 1 puff").

	Yes	No
1. Cigarettes	1	2
2. Cigars	1	2
3. Pipe (with tobacco)	1	2
4. Little cigars, cigarillos, bidis (like Black & Milds, Swisher Sweets, Phillies Blunt, or Captain Black)	1	2
5. Electronic cigarettes/vaping devices (like vape pens or e-hookah, NOT used for marijuana)	1	2
6. Chewing tobacco (like Red Man, or Beech Nut)	1	2
7. Dip/snuff (like Skoal or Copenhagen)	1	2
8. Snus (like Camel Snus)	1	2
9. Hookah	1	2

LYAC-R B2. In the past 30 days have you used …?

	Yes	No
1. Cigarettes	1	2
2. Cigars	1	2
3. Pipe (with tobacco)	1	2
4. Little cigars, cigarillos, bidis (like Black & Milds, Swisher Sweets, Phillies Blunt, or Captain Black)	1	2
5. Electronic cigarettes/vaping devices (like vape pens or e-hookah, NOT used for marijuana)	1	2
6. Chewing tobacco (like Red Man, or Beech Nut)	1	2
7. Dip/snuff (like Skoal or Copenhagen)	1	2
8. Snus (like Camel Snus)	1	2
9. Hookah	1	2

B3. [Repeat for each product ever used B1] Are the [tobacco product fill B1=1] you typically used…

1. Menthol
2. Non-Menthol ('tobacco flavor')
3. Any other flavor (like candy, fruit, alcohol, or clove)

CPS-TUS B4. Have you smoked at least 100 cigarettes in your life? [If B1a=1 or B2a=1]

1. Yes
2. No

TCME B5. What brand of cigarettes [do/did] you [usually/last] smoke? Please select from the choices below. [If B1a=1 or B2a=1, else skip to B6] [If B1b=1 or B1d=1 say did/last; if B2b=1 or B2d=1 say do/usually]

1. American Spirit
2. Basic
3. Benson & Hedges
4. Camel
5. Carlton
6. GPC
7. Generic
8. Kent
9. Kool
10. Marlboro
11. Merit
12. Newport
13. Pall Mall
14. Salem
15. Virginia Slims
16. Winston
17. No special brand
18. Roll your own tobacco
19. Other (please specify): ___________________________

B6. What brand of cigars/little cigars/cigarillos [do/did] you [usually/last] smoke? Please select from the choices below. [If (B1b = 1 or B1d=1) OR (B2b=1 or B2d=1), else skip to C1]

1. Backwoods
2. Black & Mild
3. Blackstone
4. Cheyenne
5. Cohiba
6. Djarum
7. Dutch Masters
8. Garcia Y Vega
9. Hav-A-Tampa
10. Macanudo
11. Phillies
12. Prime Time
13. Romeo y Julieta
14. Santa Fe
15. Swisher Sweets
16. White Owl
17. Zig Zag
18. Other (please specify): ___________________________

LYAC-R B7. Which of the following best describes how you think of yourself?

1. Smoker
2. Social smoker
3. Occasional smoker
4. Ex-smoker

5. Someone who tried smoking
6. Non-smoker

ESSI-R B8. Have you ever been curious about smoking a cigarette?
1. Definitely yes
2. Probably yes
3. Probably not
4. Definitely not

ESSI-R B9. Do you think that in the future you might experiment with cigarettes?
1. Definitely yes
2. Probably yes
3. Probably not
4. Definitely not

ESSI-R B10. At any time in the next year do you think you will smoke a cigarette?
1. Definitely yes
2. Probably yes
3. Probably not
4. Definitely not

ESSI-R B11. If one your best friends were to offer you a cigarette, would you smoke it?
1. Definitely yes
2. Probably yes
3. Probably not
4. Definitely not

C. Peer influence

TruthMM C1. How many of your 4 closest friends smoke cigarettes?

1. 1
2. 2
3. 3
4. 4
5. None of my 4 closest friends smoke cigarettes

TruthMM C2. How much do you agree or disagree with the following?

	Strongly Disagree	Disagree	Neither Agree/ Disagree	Agree	Strongly Agree
It’s not a big deal if my friends smoke	1	2	3	4	5
My close friends would disapprove of me smoking occasionally at a party or other social setting.	1	2	3	4	5

D. Store Visiting and Tobacco Advertising

STORE-R D1. How often do you go to…?

	Almost Every day	Two or Three Times a Week	Once a Week	Two or Three times a month	Once a month	Less than once a month	Never
Convenience Stores (including gas stations)	1	2	3	4	5	6	7
Small markets	1	2	3	4	5	6	7
Liquor stores	1	2	3	4	5	6	7
Pharmacies (like Rite Aid or Walgreens, do not include CVS)	1	2	3	4	5	6	7
Supermarkets/ Grocery stores (like Harris Teeter or Safeway; do not include Whole Foods or Trader Joes)	1	2	3	4	5	6	7
Superstores or Warehouse clubs (like Walmart or Costco; do not include Target)	1	2	3	4	5	6	7
Tobacco stores	1	2	3	4	5	6	7
Vape shops	1	2	3	4	5	6	7
Hookah bars/lounges	1	2	3	4	5	6	7

TCME-R D2. The following list consists of different sorts of advertising, publicity, promotions, and events that are sometimes organized, conducted or sponsored by the tobacco industry.

In the **past 30 days**, how often do you remember seeing or hearing about…

	Never	Rarely	Sometimes	Frequently
a. Local community events involving the tobacco industry?	1	2	3	4
b. TV ads made by the tobacco industry?	1	2	3	4
c. Magazine or newspaper ads for tobacco products?	1	2	3	4
d. Sporting events sponsored by the tobacco industry?	1	2	3	4
e. Live music/nightclub/bar events sponsored by the tobacco industry?	1	2	3	4
f. Posters/promotions for tobacco products in local stores or supermarkets?	1	2	3	4
g. Free tobacco product give-aways (like free cigarettes)?	1	2	3	4
h. Free coupons for tobacco products?	1	2	3	4

You have reached the end of the survey.
